# Human Gingival Integration-Free iPSCs; a Source for MSC-Like Cell

**DOI:** 10.3390/ijms160613633

**Published:** 2015-06-15

**Authors:** Yasuyuki Umezaki, Yoshiya Hashimoto, Naoki Nishishita, Shin Kawamata, Shunsuke Baba

**Affiliations:** 1 Department of Oral Implantology, Osaka Dental University, Hirakata, Osaka 573-1121, Japan; E-Mail: baba-s@cc.osaka-dent.ac.jp; 2 Department of Biomaterials, Osaka Dental University, Hirakata, Osaka 573-1121, Japan; E-Mail: yoshiya@cc.osaka-dent.ac.jp; 3 Department of Bioresources for Drug Discovery, National Institute of Biomedical Innovation, Health and Nutrition, Ibaraki, Osaka 567-0085, Japan; E-Mail: nnishishita@nibiohn.go.jp; 4 Research and Development Center for Cell Therapy, Foundation for Biomedical Research and Innovation, Kobe, Hyougo 650-0047, Japan; E-Mail: kawamata@fbri.org

**Keywords:** human gingival tissues, episomal vectors, mesenchymal, plasmid, pluripotent, gingival fibroblasts

## Abstract

Mesenchymal stem cells (MSCs) are considered a potential autologous therapy for tissue engineering. The available procedures for MSC retrieval from patients are invasive, and their limited *in vitro* proliferation restricts their use in the treatment of damaged tissues. Therefore, it is important to establish an alternative and safe source of MSCs. The objective of this study was to demonstrate induced pluripotent stem cell (iPSC) generation from a combination of an accessible source tissue and an integration-free method; we also attempted the differentiation of iPSCs into MSC-like cells (MSLCs) for future autologous tissue engineering. iPSCs were derived from human gingival tissues, which are easily accessible in the field of dentistry, via the use of non-integrating episomal plasmids. Established iPSCs expressed embryonic stem (ES) cell-specific markers, as assessed by gene analysis and immunocytochemistry. Embryoid bodies and teratoma formation were formed from iPSCs, showing their capacity to differentiate into three germ layers. Furthermore, we were successful in differentiating iPSCs into MSLCs. They tested positively for their capacity of trilineage differentiation. Our results demonstrate that human gingival integration-free iPSCs, readily accessible stem cells generated using episomal plasmid vectors, are a promising source of MSLCs, which can be used in tissue regeneration.

## 1. Introduction

Mesenchymal stromal/stem cells (MSCs) are heterogeneous cells that possess multilineage differentiation potential [1]. MSCs are among the most promising adult stem cells for clinical applications and mainly isolated from bone marrow, adipose tissue, and various dental tissues [2,3]. However, it is known that MSC proliferation capacity decreases with the donor age [4]. Furthermore, MSCs have a limited capacity to proliferate *in vitro*, making it very difficult to acquire sufficient cell numbers for implantation [5]. The available procedures for MSC retrieval from patients are invasive and labor intensive. Therefore, there is an urgent need for an alternative source of MSCs.

Human embryonic stem cells (ESCs) are derived from the inner cell mass of mammalian blastocysts [6], which limits their clinical use by significant ethical issues. To circumvent these issues, induced pluripotent stem cells (iPSCs) were generated from human somatic cells by retroviral transduction of four transcription factors [7]. Established human iPSCs share similar characteristics with ESCs. So far, iPSCs have been established from dermal fibroblasts [7], peripheral blood [8], dental pulp cells [9,10], gingival fibroblasts [11], periodontal ligaments [12], apical papilla, oral mucosa [13], third molar mesenchymal stromal cells [14], and immature dental pulp stem cells [15].

Gingival tissues when removed during dental treatments, such as periodontal surgery and dental implants, are considered biomedical wastes [11]. In addition, gingival wounds are known to heal relatively quickly compared to skin wounds [16] and involve lesser aesthetic considerations since it is sampled from the mouth in contrast to skin. In a previous study, Egusa *et al.* reported that the reprogramming efficiency of mouse gingival fibroblasts was higher than that of dermal fibroblasts [11]. Furthermore, iPSC generation from peripheral blood requires a cell isolation process for obtaining a sufficient number of cells [8]. Such a step is costly and time-consuming compared to the simple and easy culture of human gingival fibroblasts.

Egusa *et al.* suggested that the collection of gingivae from healthy volunteers and iPSC generation from these tissues might allow the development of a cell bank for a wide range of medical applications [11]. In 2010, they successfully derived iPSCs from human gingival fibroblasts (HGFs) by retroviral transduction of transcription factors and suggested human gingiva to be one of the easily accessible tissues for future autologous iPSC therapies [11]. However, retroviral integration increases the risk of tumor formation, and an integration-free method decreases this potential risk [17]. Several integration-free methods have been reported for iPSC generation [18]. Notably, Okita *et al.* simply and effectively generated integration-free iPSCs from human dermal fibroblasts (HDFs) with episomal plasmid vectors consisting of six transcription factors [17]. For future autologous cell therapies, the accessible source tissue and integration-free method of efficient reprogramming represent an ideal combination for iPSC generation.

Recently, many groups have successfully established MSC-like cells (MSLCs) from ES/iPSCs [5,19,20,21,22]. Lian *et al.* [23] demonstrated that these cells exhibited a greater proliferative capacity than primary cultures of bone marrow-derived MSCs [5,23]. Moreover, they might not have a tumorigenic potential, making them safer for implantation into humans [23].

The objective of this study was first, to assess the generation of iPSCs from the combination of primary human gingival fibroblasts and episomal plasmid vectors; and second, to differentiate iPSCs into MSC-like cells. Such iPSCs could be a promising source of stem cells to investigate MSLC potential for future clinical applications.

## 2. Results

### 2.1. Generation of iPSCs from HGFs with Episomal Plasmid Vectors

Three lines of HGFs were established from gingiva of 70- (HGF1), 63- (HGF2), and 60-year-old (HGF3) Asian females. Homogeneous fibroblasts emerged out of gingival connective tissues one week after the start of the culture. HGFs were exponentially expanded up to 30 passages; cells were plated at 1.5 × 10^4^ cells/cm^2^. Cells were counted at each passage. The experiment was performed up to 30 passages. The calculated population doubling of HGF was approximately 90. Colonies with a flat human ESC-like morphology and non-ESC-like colonies were counted at around day 30 after HGF transfection with episomal plasmid vectors, including human POU5F1 (also known as OCT3/4), SOX2, KLF4, L-MYC, p53 shRNA, and Lin28. The colony numbers were ~81 in ESC-like colonies and ~41 in non-ESC-like colonies (Table 1). The average number of ESC-like colony, including the standard deviation, from the 16 experiments summarized in the table was 48.6 ± 24.3. The reprogramming efficiency was about 0.5%. Some colonies obtained from HGF1 cells were mechanically picked at passage 1. After several days, four ES cell-like colonies were selected and expanded. All colonies were similar to ESCs in morphology and proliferative capacity, and named HGF-iPSCs.

**Table 1:** Colony number obtained from human gingival fibroblasts (HGFs). Number of colonies per 1 × 10^5^ cells after cell reprogramming with episomal vectors. These data are obtained from 16 independent induction experiments using HGFs from three donors.

HGFs	ES-Like	Non-ES-Like
HGF1	10	5
	12	15
	19	24
	58	21
	66	32
	71	21
	23	6
	57	23
	81	41
	67	21
HGF2	13	15
	61	21
	53	31
HGF3	72	31
	54	13
	61	21

### 2.2. Characterization of HGF-iPSCs

**Figure 1:**
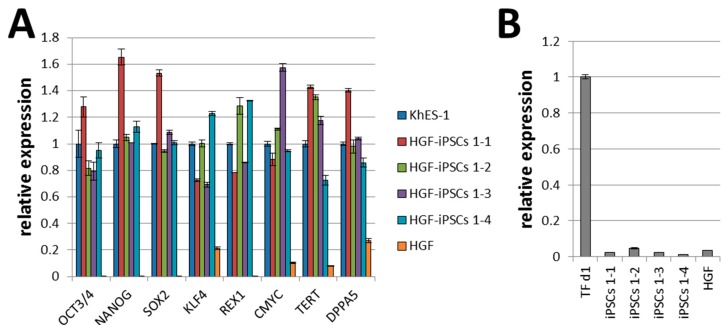

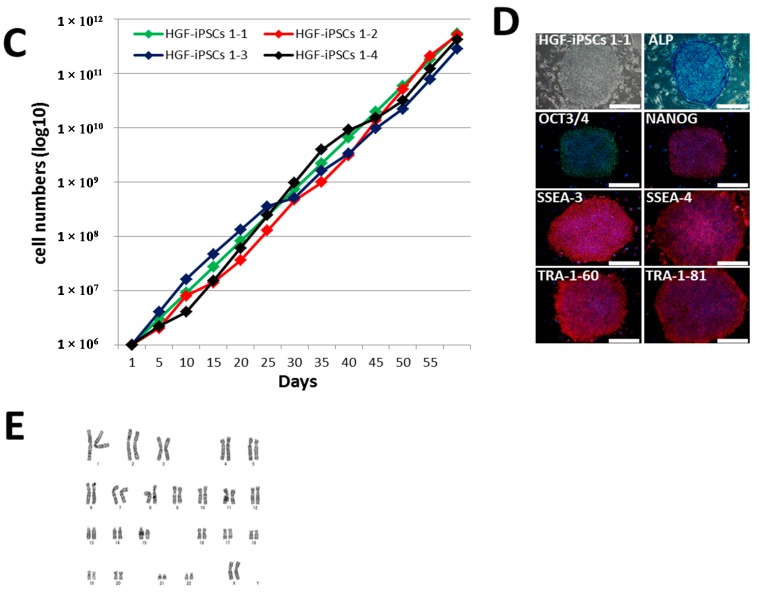
Characterization of induced pluripotent stem cells generated from human gingival fibroblasts (HGF-iPSCs). (**A**) Quantitative RT-PCR of four HGF-iPSCs, 1-1 (passage 25), 1-2 (passage 20), 1-3 (passage 21), and 1-4 (passage 20), of pluripotency-related gene expression (OCT3/4, NANOG, SOX2, KLF4, REX1, CMYC, TERT, and DPPA5). KhES-1 cells (passage 23) were used as positive control and human gingival fibroblasts (HGFs) (passage 6) as negative control were used; (**B**) Expression of episomal vector transgenes in human HGF-iPSCs, HGFs (passage 6) as negative control, and HGFs on day 1 after transduction (TF d1) with episomal vectors as positive control. QRT-PCR data represent mean ± standard deviation (SD) of three independent experiments; (**C**) Growth curve of four HGF-iPSCs; (**D**) Morphology of HGF-iPSCs 1-1 cultured on SNL feeder (passage 26). Established HGF-iPSC line 1-1 was stained to identify any ALP activities and for OCT3/4, NANOG, SSEA-3, SSEA-4, TRA-1-60, and TRA-1-81. Scale bar = 400 μm; (**E**) Karyotype analysis of iPSCs 1-1 at passage 26 by G-band staining.

Rohani *et al.* have reported that reprogramming efficiency declines with age [24]. The generation of iPSCs from elder donors was essential for future autologous cell therapies. Therefore, clones were selected from the eldest Asian female donor (70-year-old; HGF1). Four HGF-iPSCs were selected for characterization among all picked clones after 20 passages, based on their higher proliferation and stability of the ES-like morphology. HGF-iPSCs 1-1 were selected for the main figures as representative of the four lines. An expression analysis of ESC-specific and integration markers in HGF-iPSCs 1-1, 1-2, 1-3, and 1-4 was conducted using qRT-PCR. ESC-specific markers used were OCT3/4, NANOG, SOX2, KLF4, REX1, DPPA5, and hTERT, and all were expressed at similar levels than that of the ES cell line, KhES-1 (Figure 1A). Specific primers displayed the presence of the episomal vector component oriP. HGFs, as a negative control, did not display oriP, but as a positive control, HGFs day 1 after transfection (TF d1) did [25]. In contrast, no oriP was detected in any HGF-iPSCs after 20 passages (Figure 1B). These four HGF-iPSCs had not integrated the plasmids into a chromosome. These data demonstrated that the episomal vectors were spontaneously lost in the majority of HGF-iPSCs. These HGF-iPSC clones could be maintained beyond 50 passages and demonstrated ES cell-like morphology and proliferation (Figure 1C). They expressed ESC-specific surface markers, such as OCT3/4, NANOG, SSEA-3, SSEA-4, TRA-1-60, TRA-1-81, and alkaline phosphatase (Figure 1D and Figure S1). A karyotype analysis showed a normal human karyotype for all tested clones (Figure 1E and Figure S2).

**Figure 2:**
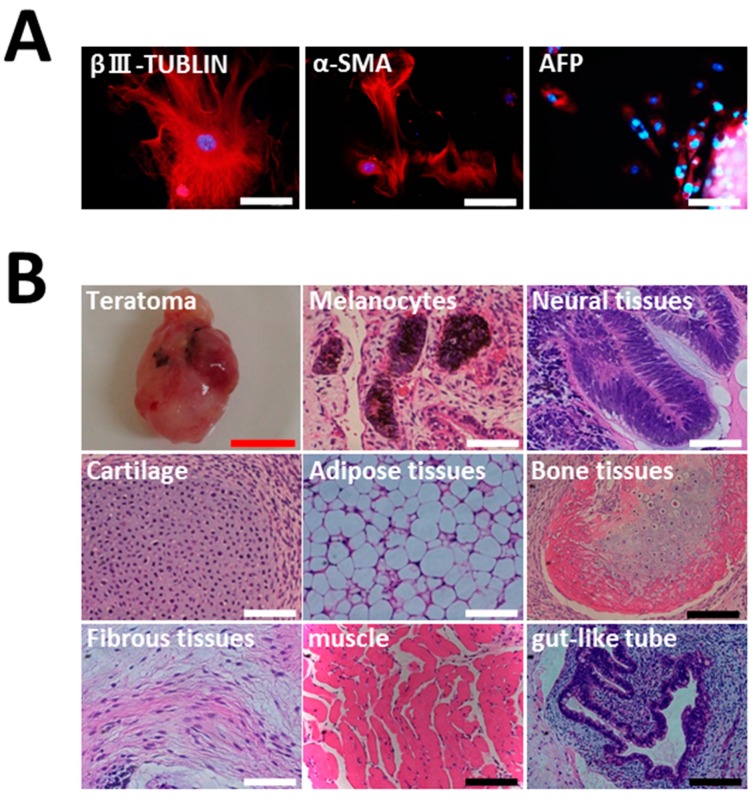
Induced PSCs have the potential to differentiate into three germ layers *in vitro* and *in vivo*. (**A**) Immunocytochemistry for β-III tubulin, α-smooth muscle actin (α-SMA), α-fetoprotein (AFP) for the differentiation capacity of HGF-iPSCs 1-1 at passage 37 into embryoid bodies; (**B**) Hematoxylin and eosin staining of teratoma derived from iPSCs 1-1 at passage 36 and observation of neural tissue (ectoderm), melanocytes (ectoderm), cartilage (mesoderm), adipose tissue (mesoderm), bone tissue (mesoderm), fibrous tissue (mesoderm), muscle (mesoderm), and gut-like tube (endoderm). White scale bar = 200 μm, red scale bar = 10 mm, black scale bar = 400 mm.

EBs were formed in order to investigate the differentiation capacity of iPSCs *in vitro*. EBs showed positive cells for β-III tubulin (ectoderm), α-SMA (mesoderm), and α-fetoprotein (AFP) (endoderm) by immunostaining (Figure 2A). Furthermore, teratoma formation assay tests *in vivo* pluripotency. Three months after injection of HGF-iPSCs under the epidermal space in the neck of immunodeficient mice, we observed tumor formation. Histological examination showed that the tumor contained various tissues, including neural tissues (ectoderm), melanocytes (ectoderm), bone tissues (mesoderm), adipose tissues (mesoderm), muscle (mesoderm), cartilage (mesoderm), fibrous tissues (mesoderm), and gut-like tube (endoderm) (Figure 2B and Figure S3). These data demonstrated that HGF-iPSCs are similar to ESCs in morphology, proliferation, surface makers, gene expression, *in vitro* differentiation, and teratoma formation.

### 2.3. Differentiation of HGF-iPSCs into MSC-Like Cells

**Figure 3:**
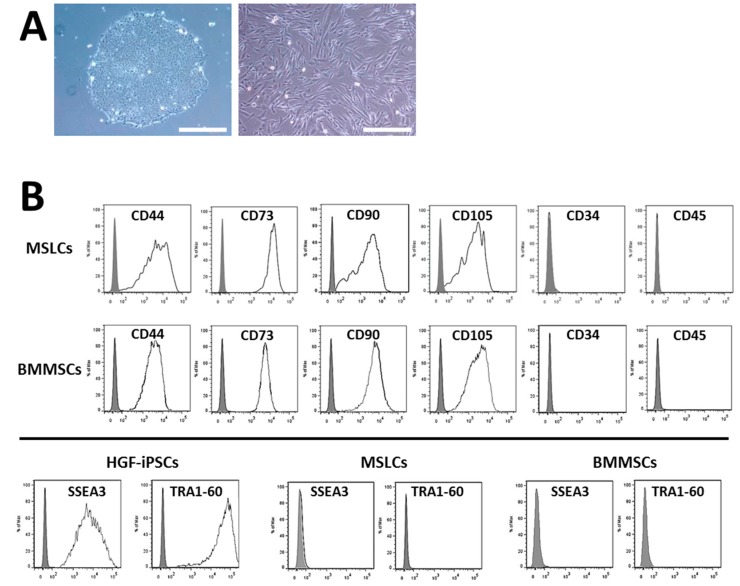

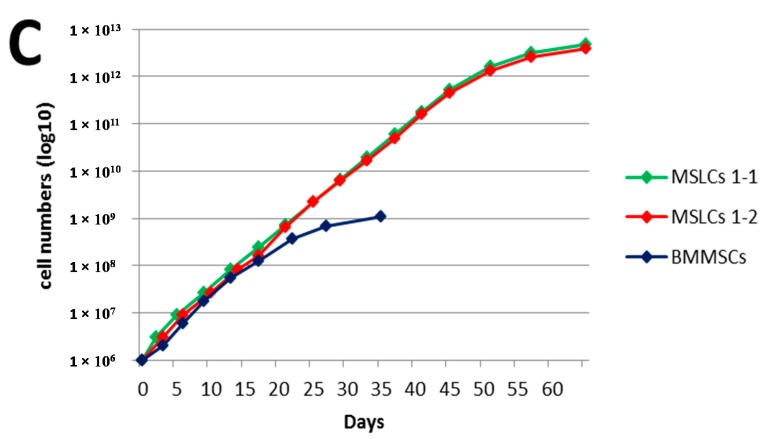
Characterization of MSC-like cells generated from iPSCs. (**A**) Morphology of HGF-iPSCs 1-1 at passage 45 cultured on GFRM with mTeSR1 (**left**). Morphology of MSC-like cells from HGF-iPSCs 1-1 differentiated (MSLCs 1-1) at passage 4 (**right**); (**B**) Flow cytometry analysis of MSC-related surface markers (CD44, CD73, CD90, and CD105), hematopoietic markers (CD34 and CD45), and pluripotent markers (SSEA3 and TRA1-60) on MSLCs 1-1 at passage 10. BMMSCs were used (passage 5) as positive control and HGF-iPSCs 1-1 (passage 45) as negative control; (**C**) MSC-like cells from HGF-iPSCs 1-2 (MSLCs 1-2) were differentiated at passage 23. Growth curve of BMMSCs, MSLCs 1-1 and 1-2. Scale bar = 400 μm. GFRM, Growth factor-reduced Matrigel.

We investigated whether HGF-iPSCs can be differentiated into MSLCs. At first, HGF-iPSCs 1-1 (passages 33, 37, and 45) and 1-2 (passage 23) were cultured in feeder-free condition for three days (Figure 3A), and direct differentiation inductions were carried out for two weeks. The differentiated iPSC morphology changed completely to a fibroblastic shape after four passages (Figure 3A). We investigated whether the fibroblastic-like cells differentiated from iPSCs were MSLCs. These cells and MSCs derived from bone marrow (BMMSCs) were analyzed by flow cytometry for human mesenchymal markers such as CD44, CD73, CD90, and CD105; endothelial and hematopoietic markers such as CD34 and CD45; and pluripotent markers such as SSEA-3 and TRA-1-60. HGF-iPSC-derived cells and BMMSCs expressed CD44, CD73, CD90, and CD105 but not CD34 and CD45 in all the passages tested. CD34 is an endothelial marker and CD45 a hematopoietic marker, both of which are not expressed in MSCs [26,27]. Therefore, we used them to distinguish MSLCs from endothelial or hematopoietic cells. In addition, HGF-iPSC-derived cells and BMMSCs did not express pluripotency markers, SSEA-3 and TRA-1-60, which HGF-iPSCs expressed (Figure 3B and Figure S4A). We confirmed that the cells could be expanded up to 15 passages (Figure 3C). Furthermore, the cells were capable of proliferating at a relatively higher rate than that of BMMSCs.

**Figure 4:**
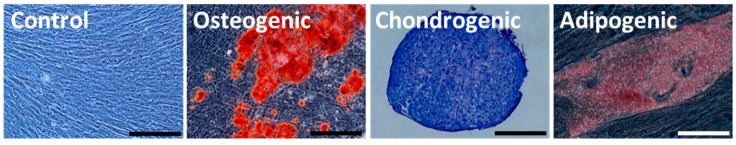
Trilineage differentiation of MSC-like cells. MSC-like cells derived from HGF-iPSCs 1-1 (MSLCs 1-1) at passage 45 were tested for their capacity of trilineage differentiation. Passage 8 MSLCs were osteogenic-, adipogenic-, and chondrogenic-differentiated for 21 days. MSLCs in control conditions were assessed for 21 days. Calcium deposition in osteogenic-differentiated MSLCs 1-1 was detected by Alizarin Red, in contrast to control conditions. Small lipid droplets in the cytoplasm of adipogenic-differentiated MSLCs 1-1 were observed by Oil Red O staining. Proteoglycan-rich extracellular matrices of chondrogenic-differentiated MSLCs 1-1 were stained red-purple by toluidine blue. Black Scale bar = 400 μm, white Scale bar = 200 μm.

Moreover, we tested the differentiation ability of MSLCs (Figure 4 and Figure S3B). For osteogenic differentiation, cells were cultured in an osteogenic differentiation medium, and calcium deposition was detected by Alizarin Red. In contrast, matrix mineralization did not occur in osteogenic-undifferentiated MSLCs when using a control medium (DMEM high glucose, 10% FBS). For adipogenic differentiation, cells were cultured in an adipogenic differentiation medium, and small lipid droplets were observed in the cytoplasm by Oil Red O staining. For chondrogenic differentiation, pellets were cultured in chondrogenic differentiation medium, and proteoglycan-rich extracellular matrix was stained red-purple by toluidine blue. These data suggested that MSLCs are similar to MSCs in some aspects, including morphology, surface markers, and trilineage differentiation.

## 3. Discussion

In the present study, we reported the generation of iPSCs from HGFs by using episomal plasmid vectors. The four established HGF-iPSC lines had similar characteristics to ESCs. In addition, after 20 passages, no plasmid had been integrated into the genome. Moreover, after transfection, we counted around 80 ESC-like colonies from 1 × 10^5^ HGFs derived from three donors. In a previous report [17], an average of 50 ESC-like colonies from 1 × 10^5^ HGFs were observed, against 30 from 1 × 10^5^ HDFs. These data suggest that the reprogramming efficiency of HGFs (0.5%) was higher than that of HDFs (0.3%) and that we managed to obtain a greater number of cells in our studies than those obtained in previous studies [17].

Based on our results, gingival tissue and integration-free reprogramming were an ideal combination for efficient iPSC generation. Induced PSCs can be maintained in feeder-free conditions for several passages, but it often results in karyotype abnormalities [28,29]. Therefore, in the next step towards clinical application, we may need to culture HGF-iPSCs on an autologous feeder layer. Cells isolated from peripheral blood do not contain fibroblasts that can be used as an autologous feeder layer. Therefore, Takahashi *et al.* [30] demonstrated that human dermal fibroblasts can be used as feeder cells for human iPSCs instead of animal feeders. In dentistry, tissues that might be used as autologous feeders include periodontal ligaments and dental pulp. However, these tissues are almost inaccessible, because periodontal ligaments need a tooth extraction and dental pulp is not regenerative. Thus, highly proliferative and more accessible HGFs could be used as autologous feeders. Therefore, further research for iPSC generation should involve the use of an autologous HGF feeder layer from gingiva. Ideally, we believe that iPSCs should be generated on autologous HGF feeder layer and episomal plasmid vectors.

In our study, MSLCs were successfully derived from iPSCs, as demonstrated by a flow cytometric profiling and trilineage differentiation; the resulting cells exhibited a higher proliferative capacity than MSCs. In previous studies, MSC-like cells from ES/iPSCs have been differentiated using various methods [5,20,21,22,26,31]. Here, we selected a simple method with trypsinization to generate MSC-like cells from iPSCs [5,26], and modified it for a feeder-free differentiation induction with growth factor-reduced Matrigel (GFRM) or Vitronectin-N (VTN-N). The change to a fibroblastic morphology and the expression of MSC markers, as determined by flow cytometry, evidenced the differentiation induction on GFRM, which occurred more rapidly than on feeder layer or VTN-N (data not shown). Differentiation was promoted rapidly by tissue culture under feeder-free conditions on GFRM. Furthermore, VTN-N is an extra-cellular matrix with a clearer composition than GFRM. Therefore, it might delay the differentiation in this protocol. Our procedure took advantage of the rapid differentiation on GFRM of iPSCs into MSLCs. Hynes *et al.* have reported that human gingiva iPSCs generated using retroviral vectors were differentiated into MSC-like cells [32]. Here, our results demonstrate that human gingival iPSCs are generated with non-integrating episomal plasmid vectors and are a promising source of MSLCs. Furthermore, the differentiation induction phase was shortened to only four passages.

In the future, *in vitro* studies of MSLCs are needed to investigate the chromosomal stability at later passages for safety testing; these studies should also involve a more comprehensive gene expression profiling and a more sophisticated differentiation capacity analysis. These results will be useful for the generation of patient-specific integration-free iPSCs and might be applicable to the generation of clinical-grade iPSCs in the future. Oral iPSCs may be of particular importance for developing innovative technologies to regenerate a missing jawbone or periodontal tissues [11]. It is known that bone and cartilage are widely present throughout the maxillofacial, including jaw, nose, ear, trachea, joint, and intervertebral disc. Once the bone and cartilage tissues are damaged due to various disorders, it becomes difficult to maintain the morphology and function of the face. Therefore, *in vivo* studies are also needed to investigate the bone or cartilage regeneration efficacy of biomaterial/MSLCs constructs.

In conclusion, our results demonstrate that integration-free iPSCs, readily accessible stem cells derived from human gingival, are a promising source of MSLCs for tissue regeneration.

## 4. Experimental Section

### 4.1. Ethics Statement

Approval for sampling human gingival tissues, establishing iPSCs, and genome/gene analysis was obtained from the Ethics Committee of Osaka Dental University (Authorization number: 110763 and 110792, approval date: 13 March 2013 and 22 November 2013) and the DNA Recombination Experiment Safety Committee of Osaka Dental University (Authorization number: 52, approval date: 13 February 2014). A written informed consent was obtained from all participants. The animal experiments followed a protocol approved by the Animal Committee of Osaka Dental University (Authorization number: 1406002, approval date: 8 July 2014).

### 4.2. Cell Culture

HGFs were established from 1 × 1 mm gingival connective tissues discarded during dental implant surgery from gingiva of 70-, 63-, and 60-year-old Asian females. Human gingiva connective tissues were placed in 35-mm tissue culture dishes and cultured in DMEM containing 10% FBS at 37 °C, 5% CO_2_ [11]. The medium was replaced every 3 days. When HGFs proliferated out of the tissues, tissues were removed. When the cells reached subconfluence, they were dissociated by 0.25% Trypsin (Invitrogen, Carlsbad, CA, USA) and transferred to 60-mm tissue culture dishes (passage 1). HGFs were regularly passaged at a 1:3 ratio every 3–4 days.

Induced PSCs were generated and maintained in ESC culture medium. These cells were cultured on mitomycin C-treated SNL76/7 (European Collection of Cell Culture (ECACC), cat. No. 07032801, lot No. 08F009). The ESC culture medium comprised DMEM-F12 medium (SIGMA, St. Louis, MO, USA) supplemented with 20% Knockout Serum Replacement (Gibco, Grand Island, NY, USA), 2 mM l-glutamine (Nacalai Tesque, Kyoto, Japan), 1% non-essential amino acid (Gibco), 0.1 mM 2-mercaptoethanol (Gibco), and 5 ng/mL FGF-2 (Reprocell, Kanagawa, Japan). For routine passaging, iPSC colonies were detached with a CTK solution (2.5 μg/mL Trypsin, 1 mg/mL Collagenase IV, 20% KSR, 1 mM CaCl_2_/PBS, 70% PBS) and split at 1:3 ratio every 4–5 days.

### 4.3. Generation of iPSCs from HGFs with Episomal Vectors

Episomal plasmids pCXLE-hOCT3/4-shp53-F, pCXLE-hSK, and pCXLE-hUL (Addgene, Cambridge, MA, USA) were mixed at 1 μg each [17]. They were electroporated into 6 × 10^5^ primary HGFs (passage 6) with Amaxa™ 4D-Nucleofector™ (Lonza, Basel, Switzerland) according to the manufacturer instructions using program DT-130. These cells were harvested using 0.25% trypsin 7 days after transduction and were transferred at 1 × 10^5^ cells per 100-mm dish on an SNL feeder layer. The following day, the culture medium was replaced with ESC culture medium. Thirty days after transduction, some colonies were mechanically picked up and transferred into a 24-well plate. After several passages, ESC-like colonies were selected for further cultivation and characterization.

### 4.4. Quantitative RT-PCR

Total RNA of all samples was extracted by RNeasy micro kit (QIAGEN, Limburg, The Netherlands). KhES-1 RNA was provided by the Foundation for Biomedical Research and Innovation. A total of 500 ng RNA (DNase-treated) was reverse transcribed into cDNA using PrimeScript^®^ RT Master Mix (Takara, Shiga, Japan). QRT-PCR was carried out in triplicates using SYBR^®^ Select Master Mix (Life Technologies, Grand island, NY, USA) in StepOnePlus™ (Life Technologies) with the following PCR program: 95 °C for 10 min, 40 cycles at 95 °C for 15 s, 60 °C for 1 min, and 72 °C for 15 s. The specific primers are listed in Table S1. Relative quantification was calculated using the 2^−ΔΔ*C*t^ method after normalization to the glycelaldehyde-3-phosphate dehydrogenase (GAPDH).

### 4.5. Alkaline Phosphatase Staining and Immunocytochemistry

Alkaline phosphatase staining was carried out using alkaline phosphatase substrate kit III (vector). For immunocytochemistry, cells were fixed for 30 min in 4% paraformaldehyde at 4 °C, followed by washing in PBS. The cells were permeabilized for 15 min with 2% bovine serum albumin and 0.1% TritonX-100 (Sigma-Aldrich, St. Louis, MO, USA) and incubated overnight at 4 °C with the primary antibodies diluted in PBS containing 2% bovine serum albumin. The cells were then washed and incubated for 1 h with the appropriate Alexa-conjugated secondary antibodies. Primary antibodies and secondary antibodies are listed in Table S2. The staining images were acquired by Olympus IX71 (OLYMPUS, Tokyo, Japan).

### 4.6. In Vitro Differentiation of iPSCs

For embryoid body (EB) formation, iPSCs were detached and collected with CTK solution, and cell clumps were transferred into a low attachment 6-well plate (Corning, Corning, NY, USA) containing DMEM-F12 based Medium (SIGMA) supplemented with 20% Knockout Serum Replacement (Gibco), 2 mM l-glutamine (Nacalai Tesque), 1% non-essential amino acid (Gibco), and 0.1 mM 2-mercaptoethanol (Gibco). After 7 days in suspension, EBs were transferred onto gelatin-coated plates and cultured for an additional 7 days in the same medium. The medium was changed every 3 days.

### 4.7. Teratoma Assay

Animal studies were approved by the Animal Committee of Osaka Dental University. For teratoma formation assay, iPSCs (1 × 10^6^) were transplanted under the epidermal space of the neck of NOD.Cg-Prkdcscid I12rgtm1Wjl/SzJ mice (The Jackson Laboratory, Bar Harbor, ME, USA). Two hundred microliters of saline was injected into another epidermal space as a negative control. All the mice developed teratomas at the injection site after 3 months. Teratomas were fixed with 4% formalin (Wako, Osaka, Japan) and stained with hematoxylin and eosin by the Business Support Center for Biomedical Research Activities.

### 4.8. Differentiation of HGF-iPSCs into MSC-Like Cells

HGF-iPSCs were cultured in mTeSR1 (Stemcell Technologies, Vancouver, BC, Canada) on growth factor-reduced Matrigel (GFRM; BD) without feeders for 3 days [19]. The medium was replaced with DMEM low glucose (Gibco), containing 10% FBS (Gibco), 10 ng/mL bFGF (PeproTech, Rocky Hill, NJ, USA) and changed every 2 days. Cells were passaged with 0.025% trypsin after 2 weeks and transferred in the same medium on a gelatin-coated plate. After passaging four times, the cell morphology changed to a fibroblastic shape. The passage was carried out every 3–5 days at a split ratio of 1:3 using 0.025% trypsin. MSCs derived from bone marrow (BMMSCs) purchased from Lonza Walkersville, Inc., were used as a control (Product No PT-2501; Walkersville, MD, USA) and cultured as previous reported [33].

### 4.9. Surface Antigen Analysis for MSC-Like Cells

Cells (5 × 10^5^) were obtained after treatment with 0.025% Trypsin (Life Technologies). Cell surface antigen staining was performed in PBS with 2% human serum albumin (Mitsubishi-Tanabe Pharmaceuticals, Osaka, Japan). The cell suspension was incubated with specific antibodies for 30 min at 4 °C. Murine anti-human antibodies were used at the recommended concentrations. Primary antibodies and isotype controls are listed in Table S2. The stained cells were analyzed with FACS Aria II (BD), and the data were analyzed using the FlowJo software (Tree Star, Ashland, OR, USA).

### 4.10. In Vitro Differentiation of MSC-Like Cells

Trilineage differentiation was carried out as previously described [5]. Osteogenic differentiation induction was performed in the following medium: DMEM-high glucose (Invitrogen) supplemented with 10% FBS (Gibco), 290 nM ascorbic acid-2-phosphate (Merck, Hessen, Germany), 5 mM β-glycerophosphate (Sigma), and 100 nM dexamethasone (Sigma). Adipogenic differentiation induction was performed in the following medium: DMEM-high glucose (Invitrogen) supplemented with 15% Horse Serum (SIGMA), and 100 nM dexamethasone (Sigma). Chondrogenic differentiation induction was performed as follows: Cells were collected in a 1.5 mL tube by centrifugation and differentiated in a medium of DMEM-high glucose (Invitrogen), supplemented with 50 mg/mL ascorbic acid, 100 nM dexamethasone, 1:100 ITS premix (BD Biosciences, San Jose, CA, USA), 40 μg/mL l-proline (Sigma-Aldrich), and 10 ng/mL human recombinant TGFβ3 (PeproTech). During all differentiation inductions, the medium was changed every 3 days, and the differentiation evaluated after 21 days by alizarin red staining, oil red staining, and toluidine blue staining [5].

### 4.11. Cell Proliferation Assessment

Cells were initially seeded at 1.5 × 10^4^ cells/cm^2^. Upon subconfluent growth to a density of 80%, cells were trypsinized and replated at the initial density. Growth curve and population doubling time were calculated by cell count.

### 4.12. Karyotype Analysis

Chromosome G-band analysis was performed at the Nihon Gene Research Laboratories, Sendai, Japan. At least 15 metaphases were analyzed.

## 5. Conclusions

In this study, first, we assessed the generation of iPSCs from the combination of primary human gingival fibroblasts and episomal plasmid vectors; and second, differentiated iPSCs into MSC-like cells (MSLCs). Establishment of iPSCs obtained from human gingival tissues, which are discarded during general dental treatments, involved the use of episomal plasmids consisting of six pluripotent factors. Furthermore, we were successful in differentiating iPSCs into MSLCs, which were expanded up to 15 passages and expressed MSC surface markers. They tested positively for their capacity for trilineage differentiation into osteogenic, adipogenic, and chondrogenic cells.

Here, our results demonstrate that human gingival iPSCs are generated with non-integrating episomal plasmid vectors and are a promising source of MSLCs. Such iPSCs could be a promising source of readily accessible stem cells to investigate MSLCs potential for future clinical applications.

## Supplementary Materials

Supplementary materials can be found at http://www.mdpi.com/1422-0067/16/06/13633/s1.
