# Supplementary material for: Human Gingival Integration-Free iPSCs; a Source for MSC-Like Cell
Source: Int J Mol Sci. 2015 Jun 15;16(6):13633–48. doi: 10.3390/ijms160613633 (PMC4490513; doi:10.3390/ijms160613633)
Supplement: Supplementary file 1 [file ijms-16-13633-s001.pdf]

Supplementary Information

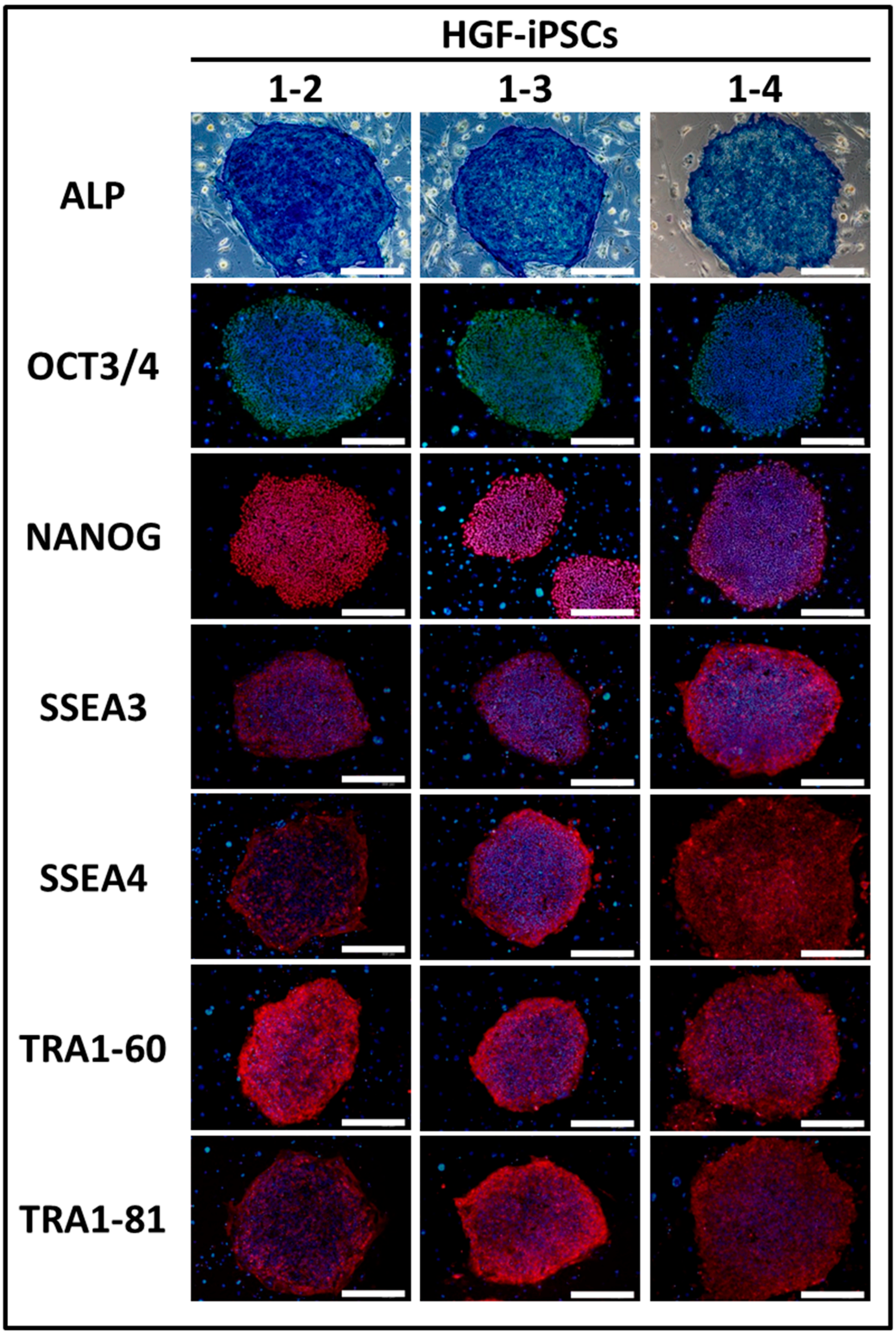

Figure S1. Characterization of the established iPSC 1-2, 1-3, and 1-4 lines from HGFs. HGF-iPSCs 1-2 (passage 23), 1-3 (passage 21), and 1-4 (passage 23), cultured on SNL feeder, were stained to identify any ALP activities and for OCT3/4, NANOG, SSEA-3, SSEA-4, TRA-1-60, and TRA-1-81. Scale bar = 400 μm.

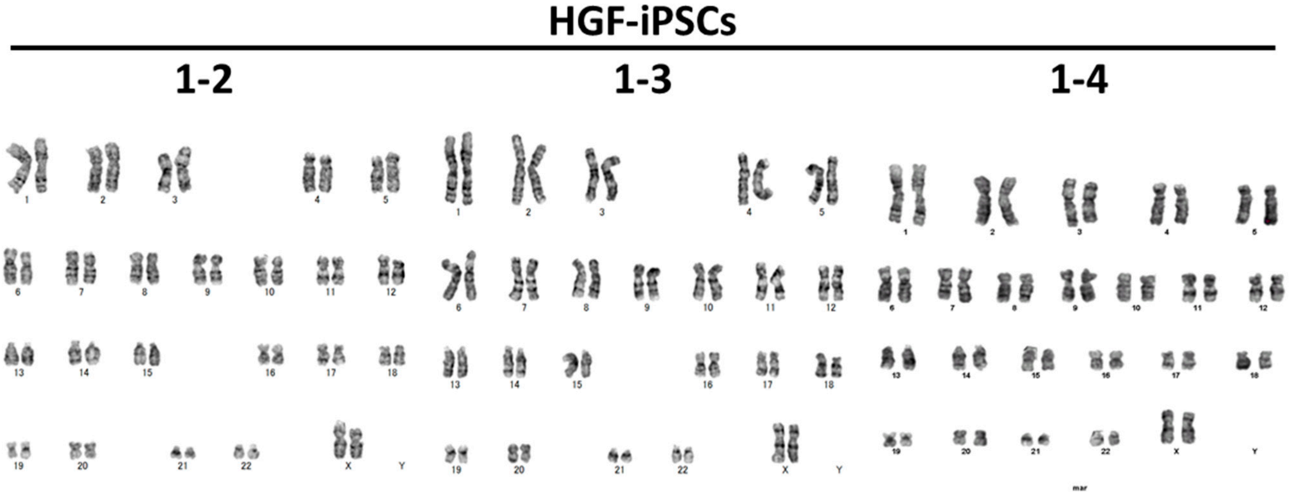

Figure S2. Karyotype analysis of the established iPSC 1-2, 1-3, and 1-4 lines from HGFs. HGF-iPSCs 1-2 (passage 23), 1-3 (passage 21), and 1-4 (passage 23), cultured on SNL feeder, were analized by G-band staining. A karyotype analysis showed a normal human karyotype for the tested three clones.

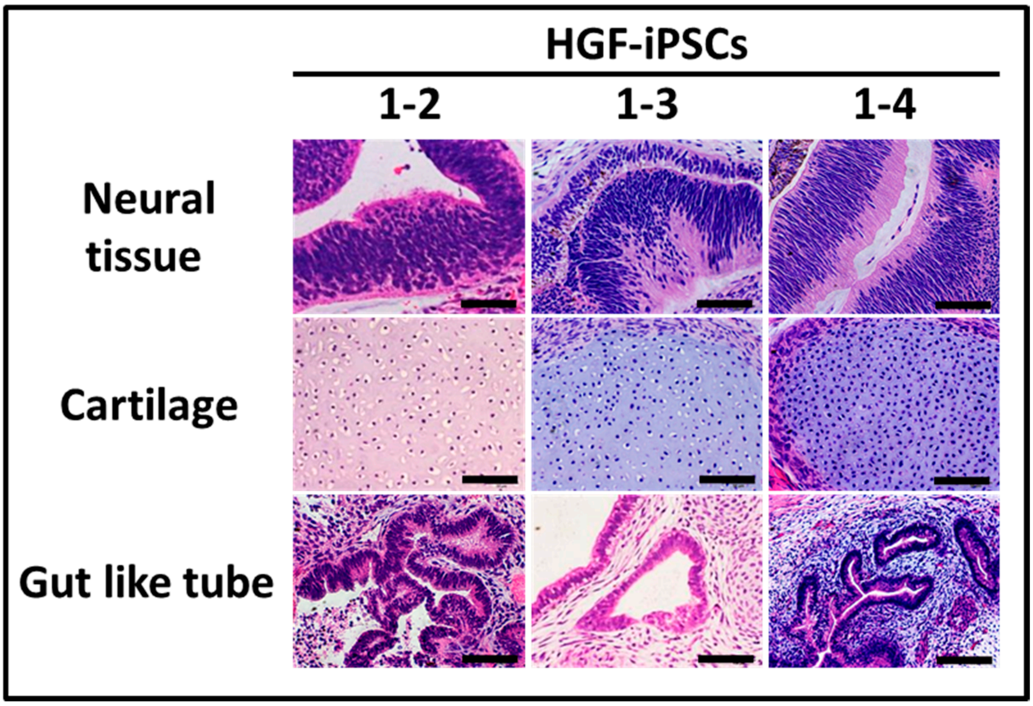

Figure S3. iPSCs have the potential to differentiate into three germ layers *in vivo*. Hematoxylin and eosin staining of teratoma derived from HGF-iPSCs 1-2 (passage 23), 1-3 (passage 21), and 1-4 (passage 23); and observation of neural tissue (ectoderm), cartilage (mesoderm), and gut-like tube (endoderm). Scale bar = 200 μm.

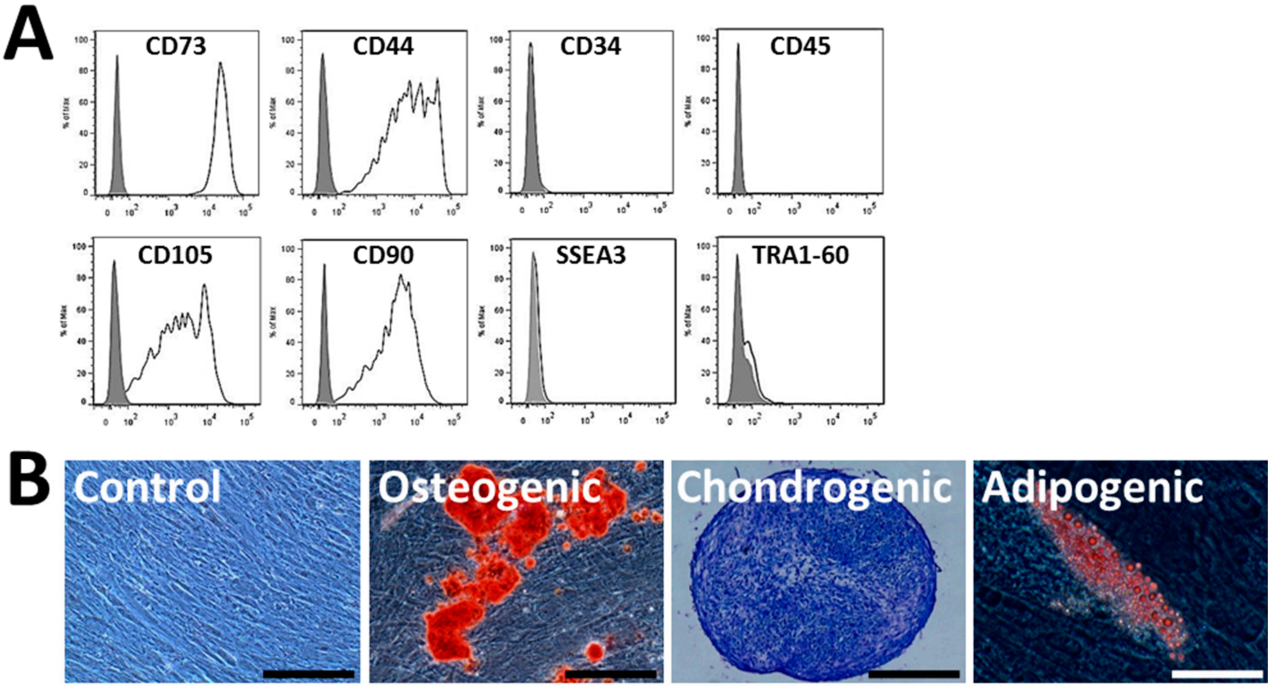

Figure S4. Characterization of MSC-like cells derived from HGF-iPSCs 1-2. (**A**) MSLCs 1-2 were differentiated at passage 23. Flow cytometry analysis of MSC-related surface markers (CD44, CD73, CD90, and CD105), hematopoietic markers (CD34 and CD45), and pluripotent markers (SSEA-3 and TRA-1-60) in MSLCs 1-2 at passage 10; (**B**) MSLCs 1-2 at passage 10 were tested for their capacity of trilineage differentiation. MSLCs in control conditions were assessed for 21 days. Calcium deposition in osteogenic-differentiated MSLCs 1-2 was detected by Alizarin Red, in contrast control condition. Small lipid droplets in the cytoplasm of adipogenic-differentiated MSLCs 1-2 were observed by Oil Red O staining. Proteoglycan-rich extracellular matrices of chondrogenic-differentiated MSLCs 1-2 were stained red-purple by toluidine blue. Black Scale bar = 400 μm, White Scale bar = 200 μm.

Table S1. List of primers used for quantitative RT-PCR of iPSCs [7,15].

Primer	Gene		Sequences (5′ to 3′)
pluripotent marker	OCT3/4	Forward	GAAACCCACACTGCAGCAGA
		Reverse	TCGCTTGCCCTTCTGGCG
	NANOG	Forward	CTCAGCTACAAACAGGTGAAGAC
		Reverse	TCCCTGGTGGTAGGAAGAGTAAA
	SOX2	Forward	GGGAAATGGGAGGGGTGCAAAAGAGG
		Reverse	TTGCGTGAGTGTGGATGGGATTGGTG
	KLF4	Forward	CGCTCCATTACCAAGAGCTCAT
		Reverse	CGATCGTCTTCCCCTCTTTG
	REX1	Forward	TGCAGGCGGAAATAGAACCT
		Reverse	TCATAGCACACATAGCCATCACAT
	TERT	Forward	CGTACAGGTTTCACGCATGTG
		Reverse	ATGACGCGCAGGAAAAATGT
	C-MYC	Forward	GTTGGTCAGGCTGGTCTTGAA
		Reverse	CATGCGCCTGTAATCCTAGCA
	DPPA5	Forward	CAGACGCGGCTGCTGAA
		Reverse	TGCTCGATGTAAGGGATTCGA
integration anarysis marker	pEP4-S	Forward	TTCCACGAGGGTAGTGAACC
		Reverse	TCGGGGGTGTTAGAGACAAC
internal control	GAPDH	Forward	CCACTCCTCCACCTTTGACG
		Reverse	ATGAGGTCCACCACCCTGTT

Table S2. Antibodies used for immunochemical staining for HGF-iPSCs and flow cytometry for MSC-like cells derived from HGF-iPSCs [7,26].

Antibodies	Supplier	Cat. No.	Dilution
OCT3/4	Santacruze	SC5279	1/200
NANOG	Cell Signaling	3580	1/800
SSEA3	abcam	ab16286	1/200
SSEA4	Millipore	MAB4360	1/200
TRA1-60	Millipore	MAB4304	1/200
TRA1-81	Millipore	MAB4381	1/200
β-ⅢTUBLIN	SIGMA	T4026	1/200
α-SMA	SIGMA	A2547	1/100
AFP	Millipore	MAB5386	1/100
DAPI	invitrogen	D1306	5 μg/mL
Alexa Fluor 488 mouse	invitrogen	A11059	1/500
Alexa Fluor 594 mouse	invitrogen	A11062	1/500
Alexa Fluor 488 rat	invitrogen	A11006	1/500
Alexa Fluor 594 rat	invitrogen	A21211	1/500
Alexa Fluor 488 rabbit	invitrogen	A11008	1/500
Alexa Fluor 594 rabbit	invitrogen	A11012	1/500
CD34	Biolegend	343606	1/100
CD44	BD	560890	1/125
CD45	Biolegend	304012	1/100
CD73	eBioscience	17-0739-42	1/65
CD90	BD	559869	1/50
CD105	eBioscience	12-1057-42	1/100
IgG1k(PE)	BD	555749	1/100
IgG1k(APC)	BD	550854	1/100
